# Uterocervical Angle (UCA) Combined With the Bishop Score as a Predictor for Successful Induction of Labor in Term Pregnancy: A Prospective Observational Study

**DOI:** 10.7759/cureus.116268

**Published:** 2026-09-15

**Authors:** Balaram Pradhan, Vina Kumari

**Affiliations:** 1 Obstetrics and Gynaecology, Tata Main Hospital, Jamshedpur, IND

**Keywords:** active phase, bishop score, cervical length, induction of labor (iol), latent phase, transvaginal ultrasound, uterocervical angle

## Abstract

Background: Traditionally, the Bishop score has been used to evaluate cervical maturity and predict the outcome of induction, but now uterocervical angle (UCA) and cervical length have gained attention to determine the possibility of successful induction of labor (IOL).

Aim and objective: This study aimed to evaluate the accuracy of ultrasonographically measured UCA combined with the Bishop score in speculating successful IOL in term pregnancy.

Methods: The study included 356 pregnant women presenting at term and undergoing IOL following institutional protocol. The pre-induction cervical status was evaluated using both the Bishop score and two-dimensional transvaginal sonography to measure cervical length and anterior UCA. Cervical assessment parameters, UCA, cervical length, and Bishop score, were compared between those with successful induction and failed IOL.

Results and conclusion: The best cut-off value in speculating the probability of successful IOL for UCA was ≥107.25° (sensitivity of 57.3% and specificity of 72.8%) and for the Bishop score was ≥4 (sensitivity of 59.3% and specificity of 72.8%). Better performance was shown by the criteria of UCA ≥107.25° or Bishop score ≥4, with 80.6% sensitivity and 64.1% specificity in speculating successful IOL. The mean duration of the latent phase and active phase were significantly shorter in patients with wider UCA (≥107.25°) (p<0.05) who entered the active phase of labor. UCA has a significant role in speculating the probability of successful IOL.

## Introduction

Induction of labor (IOL) is the initiation of uterine contractions and cervical dilation by any method (pharmacological/non-pharmacological) for vaginal delivery [1]. In India, 5-22% of patients undergo IOL out of all labor room admissions [2-4]. As is known, dilation, effacement, station, cervical consistency, and position are the factors that define the Bishop score [5]. The traditional method to assess cervical status is digital examination, summarized in the modified Bishop score [5]. However, several studies have commented on its limitations, including its high inter- and intra-observer inconsistency and its 23-64% sensitivity [6]. Few recent studies have shown better sensitivity of transvaginal sonography (TVS) and avoid inter-observer variations with better reproducibility [7-9].

Uterocervical angle (UCA) is formed between the endocervical canal and the lower edge of the anterior uterine wall [10]. The resultant vector force derived from the anterior wall of the uterus and cervical canal leads to a more linear force on the cervical canal, ultimately leading to the successful IOL [11]. It has been seen in some studies that patients with wider UCAs were more likely to have a short-lived active phase.

Given the potential but limited evidence on the role of UCA in predicting successful IOL in term pregnancies, there is a dearth of literature investigating the same among the Indian pregnant population.

## Materials and methods

This prospective observational study was carried out in the Department of Obstetrics and Gynaecology at Tata Main Hospital in Jamshedpur, India, from June 2022 to May 2024. The study was reported as per the Strengthening the Reporting of Observational Studies in Epidemiology (STROBE) guidelines. Data collection was initiated after obtaining approval from the Institutional Ethics Committee of Tata Main Hospital, Jamshedpur (approval number: TMH/IEC/JUNE/038/22). The study included 356 pregnant women presenting at term and undergoing IOL following institutional protocol. Pregnant women with a gestation period of more than 37 weeks, a singleton pregnancy with vertex presentation, and women having a Bishop score of less than 6, a post-dated pregnancy, gestational hypertension, gestational diabetes, and intrahepatic cholestasis of pregnancy were included in the study. Women who refused to participate in the study, women with multiple pregnancies, malpresentation, previous uterine scar (caesarean section, hysterotomy, or myomectomy), pre-labor rupture of membranes, fetal distress, previous cervical surgeries, or a Bishop score of more than 6, and women who were already in labor were excluded from the study.

Method of data collection

All pregnant women with a gestational age of more than 37 weeks with singleton vertex pregnancy who presented to the labor room were screened for their eligibility. Ensuring anonymity and confidentiality of the data provided by them, the patients were informed of the purpose and procedure of the study, and written informed consent was subsequently obtained. The cervix was assessed using the Bishop score and two-dimensional (2D) TVS. The Bishop score is calculated by cervical dilation, effacement, fetal station, consistency, and position of the cervix and is open access and freely available for clinical and academic reproduction [5]. TVS was conducted with a 7 MHz probe (GE Voluson P8, GE HealthCare, Chicago, IL, USA). Cervical assessment using the Bishop score was done by the doctor on duty in the labor room. Both the examiner and sonographer were blinded to each other's findings.

To measure the UCA, a first line was drawn from the internal os to the external os. A second line was then drawn to mark the lower uterine segment, and the line was extended further to meet the first line. The UCA was the angle formed by the two lines (see Figure 1). IOL was carried out as per the standard institutional protocol, as per the National Institute for Health and Care Excellence (NICE) guidelines [10]. Prostaglandin E2 (PGE2) gel was placed in the cervical canal every six hours for three doses. Subsequent doses were withheld if the patient was in active labor, there was rupture of the membrane, or the cervix was ≥3 dilated or >60% effaced, with 2-3 uterine contractions within 10 minutes. Any pregnant woman with non-reassuring cardiotocography (CTG) findings or who developed any evidence of fetal distress was excluded from the study and was delivered by caesarean section. If induction was not successful with three cycles of dinoprostone, caesarean delivery was conducted.

**Figure 1:**
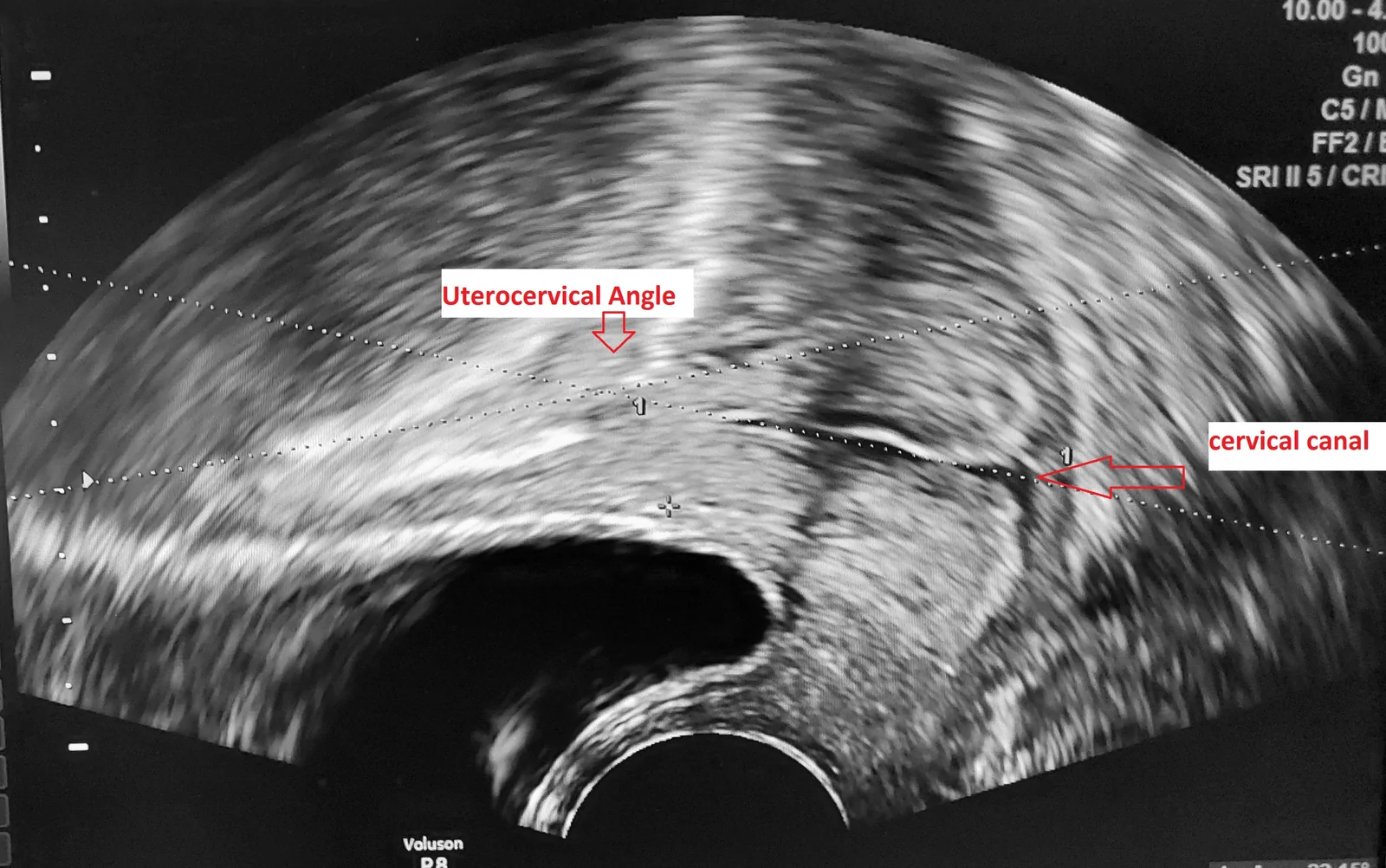
Uterocervical angle

Successful IOL was defined as follows: if the patient entered into the active phase of labor (>5 cm cervical dilation). In contrast, those who did not enter the active phase of labor (marked by failure to achieve regular uterine contractions even after the insertion of three intracervical PGE2 gels at six-hour intervals and 12 hours of oxytocin administration after rupture of the membranes) were considered as failed labor induction, which was an indication for caesarean section [5].

Failure to progress was defined as follows: no cervical dilation during the active phase of labor (≥5 cm) for the last two hours and no descent of the fetal head during the second stage of labor for at least one hour despite adequate uterine contractions [5]. The assembled data was tabulated in Microsoft Excel 2016 (Microsoft Corp., Redmond, WA, USA) and analyzed using IBM SPSS Statistics for Windows, V. 23.0 (IBM Corp., Armonk, NY, USA). Receiver operating characteristic (ROC) curve analysis was undertaken to identify and compare the cut-off values of UCA, cervical length, and Bishop score. A p-value of <0.05 was considered statistically significant.

## Results

Before IOL, patients with Bishop scores of 0, 1, 2, 3, 4, 5, and 6 were 2.2%, 9.8%, 25.3%, 12.6%, 11.5%, 34.3%, and 4.2%, respectively (Table 1).

**Table 1:** Distribution according to the Bishop score (N=356) Median (IQR): 3 (2-5); minimum, maximum: 0, 6 IQR: interquartile range

Bishop score	Frequency	Percentage
0	8	2.2
1	35	9.8
2	90	25.3
3	45	12.6
4	41	11.5
5	122	34.3
6	15	4.2

Before IOL, patients with cervical lengths between 3 cm and 3.99 cm were 33.2%, and patients with cervical lengths less than 2 cm were 29.2% (Table 2).

**Table 2:** Distribution according to the length of the cervix (N=356) Median (IQR): 2.3 (1.9, 3.2) cm; minimum, maximum: 0.9 cm, 3.9 cm IQR: interquartile range

Length of cervix	Frequency	Percentage
<2 cm	104	29.2
2-2.99 cm	134	37.6
3-3.99 cm	118	33.2

The proportion of participants with successful IOL, defined by entry to the active phase of labor, was 71.1%, while the remaining 28.9% of participants failed to enter the active phase of labor (Table 3).

**Table 3:** Distribution according to the outcome of IOL (N=356) IOL: induction of labor

Outcome of IOL	Frequency	Percentage
Successful IOL (entered the active phase of labor)	253	71.1
Failed IOL (did not enter the active phase of labor)	103	28.9

While 91.7% of the patients with successful IOL underwent vaginal delivery, 8.3% of them were delivered by lower segment caesarean section (LSCS). Moreover, all patients with failed IOL were also delivered by LSCS (Table 4).

**Table 4:** Distribution according to the mode of delivery and outcome of IOL (N=356) Values are represented as n (%). VD: vaginal delivery; IOL: induction of labor

Mode of delivery	Frequency (%)	Successful IOL (n=253)	Failed IOL (n=103)
VD	232 (65.2)	232 (91.7)	0 (0.0)
LSCS	124 (34.8)	21 (8.3)	103 (100.0)

On comparison of maternal characteristics between study participants with successful and failed IOL, it was observed that mean maternal age, gestational age, distribution of parity, and pre-induction cervical length were statistically comparable (p>0.05). However, pre-induction UCA and Bishop score were significantly higher among those with successful IOL as compared to those with failed IOL (p<0.001) (Table 5).

**Table 5:** Association between outcomes of IOL and important maternal characteristics and cervical parameters (N=356) *P-value was calculated using the Mann-Whitney U test (for continuous variables), and p<0.05 was considered to be statistically significant. UCA: uterocervical angle; IOL: induction of labor; IQR: interquartile range; NA: not applicable

Characteristics	Successful IOL (n=253)	Failed IOL (n=103)	P-value*	t-statistic
Maternal age; mean (±SD)	28.47 (±4.6)	28.11 (±4.7)	0.503	0.670
Gestational age at IOL; mean (±SD)	39.2 (±2.0)	39.3 (±2.1)	0.978	-0.028
Parity; n (%)				
Nulliparous	187 (73.9)	80 (77.7)	0.695	0.7277
Primiparous	49 (19.4)	16 (15.5)	NA	-
Multiparous	17 (6.7)	7 (6.8)	NA	-
Bishop score; median (IQR)	4 (2-5)	2 (2-5)	<0.001	5.117
UCA; median (IQR)	110.2 (101.35-115.45)	102.5 (99.6-112.0)	<0.001	4.571
Cervical length; median (IQR)	2.1 (1.8-2.9)	3.1 (2.0-3.5)	0.065	-2.211

The diagnostic test parameters of the UCA and Bishop score were compared for predicting successful IOL (Table 5). The odds ratio of UCA was 3.59 (95% CI: 2.18-5.93; p<0.001), and that of the Bishop score was 3.90 (95% CI: 2.36-6.44; p<0.001) (Table 6).

**Table 6:** Diagnostic test parameters of the uterocervical angle and Bishop score in predicting successful induction of labor PPV: positive predictive value; NPV: negative predictive value; OR: odds ratio

Parameters	Uterocervical angle	Bishop score
Cut-off value at maximum Youden's index	≥107.25	≥4
Sensitivity (95% CI)	57.3% (51.0-63.5)	59.3% (53.0-65.4)
Specificity (95% CI)	72.8% (63.2-81.1)	72.8% (63.2-81.1)
PPV (95% CI)	83.8% (78.8-87.9)	84.3% (79.4-88.2)
NPV (95% CI)	41.0% (36.6-45.5)	42.1% (37.6-46.8)
OR (95% CI)	3.59 (2.18-5.93)	3.90 (2.36-6.44)
P-value	<0.001	<0.001

Better performance was shown by the criteria of UCA ≥107.25° or Bishop score ≥4, with 80.6% sensitivity and 64.1% specificity in predicting successful IOL (Table 7, Table 8). The odds ratio was 7.42 (95% CI: 4.46-12.36; p<0.001).

**Table 7:** Performance of the combination of UCA and Bishop score in predicting successful IOL (N=356) Values are presented as n (%). ^#^Based on the chi-squared test for categorical data, with p<0.05 considered statistically significant. UCA: uterocervical angle; IOL: induction of labor; NS: not significant

Parameter	Total patients	Outcome of IOL		P-value^#^	Chi-squared value
		Successful IOL (n=253)	Failed IOL (n=103)		
Combined (AND)					
UCA ≥107.25° and Bishop score ≥4	110	91 (36.0)	19 (18.4)	<0.001	10.525
Otherwise	246	162 (64.0)	84 (81.6)	NS	-
Combined (OR)					
UCA ≥107.25° or Bishop score ≥4	241	204 (80.6)	37 (35.9)	<0.001	66.912
Otherwise	115	49 (19.4)	66 (64.1)	NS	-

**Table 8:** Diagnostic test parameters of the combination of UCA and Bishop score in predicting successful induction of labor The p-value was calculated using the Mann-Whitney U test, with p<0.05 considered statistically significant. PPV: positive predictive value; NPV: negative predictive value; OR: odds ratio

Parameters	Combined (AND)	Combined (OR)
Sensitivity (95% CI)	36.0% (30.05-42.22)	80.6% (75.22-85.31)
Specificity (95% CI)	81.6% (72.7-88.51)	64.1% (54.03-73.3)
PPV (95% CI)	82.7% (75.55-88.13)	84.7% (80.88-87.78)
NPV (95% CI)	34.2% (31.28-37.13)	57.4% (50.19-64.29)
OR (95% CI)	2.48 (1.42-4.34)	7.42 (4.46-12.36)
P-value	0.002	<0.001

The area under the curve (AUC) of the ROC curves for UCA and Bishop score in speculating successful IOL was 0.651 (p<0.001) and 0.643 (p<0.001), respectively (Figure 2). AUC for cervical length was 0.331 with no statistical significance (p=0.265), annulling its role in speculating successful IOL (Figure 2).

**Figure 2:**
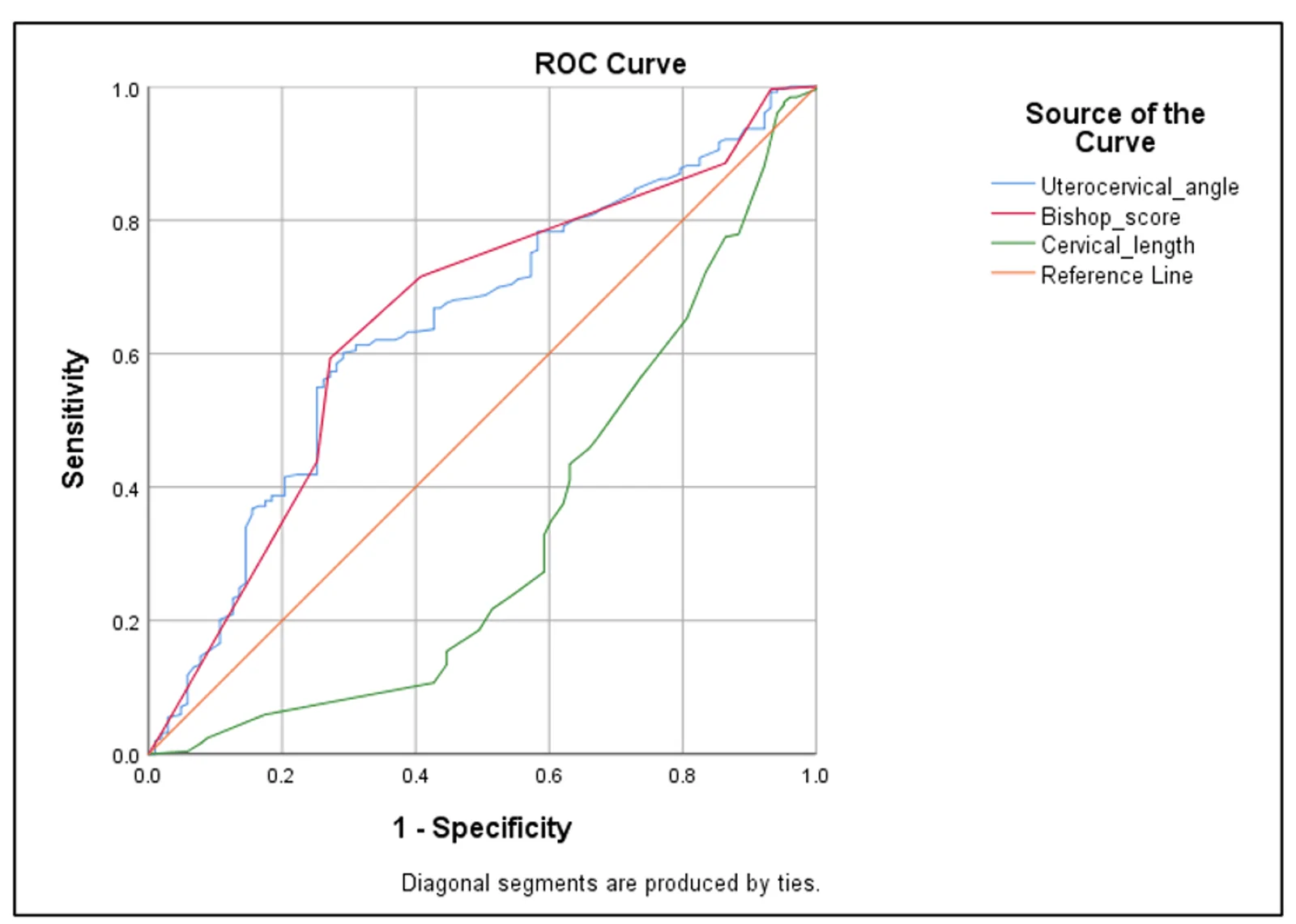
ROC curves for uterocervical angle, Bishop score, and cervical length in predicting successful induction of labor ROC: receiver operating characteristic

The mean duration of the latent phase and active phase was significantly shorter in patients with wider UCA (≥107.25°) (p<0.05) who entered the active phase of labor and underwent delivery subsequently (Table 9).

**Table 9:** Association between UCA and duration of latent phase and active phase of labor in participants with successful IOL (N=253) Values are presented as mean (±SD). *The p-value was calculated using the Mann-Whitney U test, with p<0.05 considered statistically significant. UCA: uterocervical angle; IOL: induction of labor

Characteristics	UCA ≥107.25 (n=145)	UCA <107.25 (n=108)	P-value*	U-value
Duration of latent phase (hours)	11.16 (±3.29)	12.59 (±3.88)	0.002	-3.077
Duration of active phase (hours)	4.38 (±0.78)	5.10 (±1.17)	<0.001	-5.561

## Discussion

After IOL, the increasing risks of caesarean section, perinatal asphyxia, and fetal distress have raised concern for doctors and patients. Among the various sonographic cervical parameters, the UCA, combined with the Bishop score, has recently gained attention for more accurately determining the probability of successful induction [12].

In concordance with most related studies, the present study's cervical assessment was performed before induction. In our study, the median (interquartile range (IQR)) Bishop score was 3 (2-5) (Table 1). Similar pre-induction mean Bishop scores were found in other studies. Keepanasseril et al. reported the mean Bishop score to be 4.52 (±1.57) cm [13]. In the present study, patients with a Bishop score of more than 6 were excluded because these patients usually enter into spontaneous labor.

The proportion of participants with successful IOL, defined by entry into the active phase of labor, was 71.1% (Table 3). The success rate indicates that the induction methods were generally effective, though a notable proportion (28.9%) did not progress to active labor, requiring delivery by caesarean section. Dagdeviren et al. [14] reported that although 115 out of 150 women (76.7%) had successful IOL, 15.6% of them (18 out of 115) were delivered by LSCS.

Although the Bishop score is widely accepted to assess cervical status, it has some limitations. For example, dilation, effacement, and position of the cervix vary from observer to observer and are quite subjective [7]. More recently, ultrasonographic parameters such as cervical length and UCA have captured interest in evaluating the cervical status before induction. It is hypothesized that the resultant vector force of UCA is the determining factor; the greater or wider the UCA, the more direct the force towards the cervix, leading to better cervical dilation and effacement, which leads to an increased possibility of entering the active phase of labor. In contrast, diminished force on the cervical outlet in a narrower UCA (or acute angle) leads to a reduced possibility of entering the active phase of labor. This hypothesis might possibly explain the significantly higher pre-induction UCA and Bishop score among those with successful IOL as compared to those with failed IOL (p<0.001) and the lack of statistically significant differences in pre-induction cervical length between successful IOL and failed IOL (p=0.065). A study by Abdelhafeez et al. identified the best cut-off value of UCA as 110.2° with 87% sensitivity and 93% specificity in predicting successful labor induction [15].

ROC curve analysis was done between UCA and Bishop score, and both were seen above the 45° line, suggesting a significant relationship with a higher probability of entering the active phase of labor (Figure 2). The AUC of ROC curves for UCA (0.651) and Bishop score (0.643) in predicting successful IOL was significant (p<0.001). AUC for cervical length was statistically not significant (p=0.065), nullifying its role in speculating successful IOL (Table 5).

In our study, after analyzing UCA and Bishop score combinedly, the criteria of UCA ≥107.25° and Bishop score ≥4 resulted in successful IOL. Better performance was shown by the criteria of UCA ≥107.25° or Bishop score ≥4 in the prediction of successful IOL (Table 7, Table 8). Our observations differ from that reported by Yang et al. where they stated that higher performance was demonstrated by Bishop score ≥6 in nulliparous/≥5 in multiparous and UCA >108.4°, with a sensitivity of 44.6%, a specificity of 96.0%, and a PPV of 96.2%, and Bishop score ≥6 in nulliparous/≥5 in multiparous or UCA >108.4°, with a sensitivity of 85.7%, a specificity of 50.0%, and a PPV of 78.7% [16]. Our observation showed successful IOL with a comparatively less favourable Bishop score and/or UCA cut-off value.

Among patients undergoing successful IOL, the duration of the latent phase in women with pre-induction UCA ≥107.25° was statistically shorter than that of those with pre-induction UCA <107.25° (11.16 hours vs. 12.59 hours). Also, the duration of the active phase was statistically shorter in women with wider UCA (Table 9). In a previous study, Dagdeviren et al. [14] reported successful IOL based on entering the active phase within 24 hours. In our study, a wider UCA suggested earlier active-phase entry, typically within 12 hours rather than 24 hours.

Additionally, there was no significant difference in parity distribution between the successful UCA-based IOL group and the failed group, indicating that UCA may be independent of parity. Consistent with our findings, Eser and Ozkaya [12] also identified UCA as a predictor of successful labor induction, particularly with a latent phase under 12 hours, regardless of parity. UCA, calculated using vector summation from the anterior wall and endocervical canal, differs from the Bishop score, which is based on cervical position and effacement [14].

Study limitations

Since the study was conducted at a single centre, the findings may not be generalizable beyond this specific setting. Another limitation is the potential inter- or intra-observer variation in assessing the Bishop score and UCA. While the same sonographer measured the UCA, multiple residents assessed the Bishop score, which could introduce bias and affect the performance of both predictive measures. Additionally, the low AUC for UCA made it challenging to evaluate its superiority compared to the Bishop score. Future multicentric research would help to address these limitations and provide more comprehensive and robust evidence regarding the role of UCA and the Bishop score in predicting successful IOL.

## Conclusions

The present study highlights key factors influencing the success of labor induction, particularly the importance of pre-induction UCA and Bishop scores. From our study, it can be concluded that transvaginal sonographically measured pre-induction UCA has a significant role in predicting the likelihood of successful IOL. Also, a combination of UCA ≥107.25° or Bishop score ≥4 was found to have better sensitivity and PPV in speculating successful IOL. These findings can inform clinical practices and decision-making for labor induction, aiming to optimize outcomes for both mothers and infants. Further multicentric trials are very much essential for comprehension in this regard.
